# Older Chinese and Filipino American Immigrants with Type 2 Diabetes and their Adult Child: A Qualitative Dyadic Exploration of Family Support

**DOI:** 10.1007/s10823-024-09505-w

**Published:** 2024-05-09

**Authors:** Hillary Nicole Peregrina, Maria L. G. Bayog, Adam Pagdilao, Melinda S. Bender, Therese Doan, Grace J. Yoo

**Affiliations:** 1 Department of Social Welfare, University of California, Los Angeles, Los Angeles, USA; 2 School of Nursing, University of California, San Francisco, San Francisco, USA; 3 University of California, Riverside, Riverside, USA; 4 Department of Asian American Studies, San Francisco State University, San Francisco, USA; 5 School of Nursing, San Francisco State University, San Francisco, USA

**Keywords:** Asian Americans, Family Support, Type 2 Diabetes, Elderly Immigrants, Dyadic, Qualitative

## Abstract

Type 2 Diabetes (T2D) among older Asian American immigrants (AA) is a growing concern. Asian Americans represent 9% of diagnosed diabetes. Very little is known on how older Asian American immigrants with T2D navigate diabetes management, in particular the role of family support. This qualitative study examines Chinese and Filipino Americans, the two largest Asian subgroups in the US (4.2 million, and 3.6 million, respectively), and family support dynamics among adult children and their parents diagnosed with T2D. Ten dyads (*n* = 20) made up of adult children and aging parents participated in in-depth and dyadic interviews. Results indicate that family support occurs in a trajectory of stages. The following thematic patterns emerged in these dyads around support: independence, transitions, partnership, and stepping in. The findings point to various supportive stages that Asian American adult children and aging parents with T2D experience and the importance of developing supportive interventions for both adult children and aging parents at these various stages.

## Background

In 2020, Asian Americans represented 19.9 million of the U.S. population. Older Asian Americans represent a growing Asian American sub-population. Diabetes rates increase with age, peaking at 29.2% among those 65 years or older (CDC, 2020). Older Asian American immigrants are experiencing increased type 2 diabetes (T2D) rates (Adia et al., 2020). Asian Americans are often stereotyped as the “model minority,” a prevalent and misleading assumption that depicts Asian Americans as “healthy.” Yet, Asian American (AA) adults constitute 9.5% of those diagnosed with diabetes and report poorer diabetes management, including fewer daily self-administered feet checks and fewer weekly glucose checks (Islam et al., 2013; CDC, 2020). Chinese American and Filipino Americans, the largest and fastest growing subgroups among Asian Americans, experience increasing diabetes prevalence with 12.6% among Filipino Americans, and 5.5% among Chinese Americans (Adia et al., 2020). Despite these rates, there are few studies about diabetes care of older adults and families, and almost none focused on Asian Americans, specifically Chinese and Filipino Americans.

### Diabetes Management and Family Support

Diabetes self-management includes active engagement in self-care activities to improve one’s behaviors and health outcomes (Carpenter et al., 2019). Effective diabetes management includes key elements such as (1) diabetes education, (2) exercise and weight management, (3) nutrition, (4) medication adherence, (5) stress and lifestyle modification, and (6) blood pressure regulation. Adults with T2D often receive self-management support from adult children, siblings, or close friends who live outside of their homes (Mayberry et al., 2019a). Among immigrants in the United States, factors that may impact diabetes self-management include changes in dietary lifestyle, low socioeconomic status, access to health insurance, social support resources, limited English proficiency, psychosocial status, and gender roles (Kim et al., 2023).

Previous studies report that self-management is affected directly by social support and works best when family members are in partnership with patients (Al-Dwaikat et al., 2023). For instance, family members of those with diabetes can support regular glucose testing and provide ongoing encouragement (Al-Dwikat et al., 2023; Lee et al., 2018). The most frequent self-management activities that family members assist include maintaining diet, provision of medication, and joining physician visits (David et al., 2019). Previous research demonstrates self-care management behaviors and quality of family relationships may account for variability in hemoglobin A1C (David et al., 2019). Previous research also demonstrates that emotional closeness with familial care providers is associated with medication adherence, more fruit/vegetable intake, and lower diabetes related distress (Mayberry et al., 2019b). Limited studies focused on diabetes self-management and family support among racial/ethnic minorities show that family can play a vital role in increasing health literacy and supporting the lifestyle changes necessary for managing diabetes (Hu et al., 2016).

In studies of family support and diabetes management among older Asian American immigrants, family members exhibited both supportive and unsupportive behaviors. A systematic review of family-based interventions for Chinese American immigrants with diabetes demonstrates that including family members in patients’ diabetes education and counseling sessions improved diabetes care (Li & Tong, 2023). Most studies on family support amongst older Asian Americans focus on spousal support for T2D. Among East Asian American immigrants (China, Korea, Japan, and Taiwan), spouses without diabetes exhibited unhelpful behaviors by displaying reluctance to adopt appropriate dietary modifications, thus creating conflict about who in the family should observe food restrictions (Li-Geng et al., 2020). Among Korean immigrants with T2D, poor self-management, more diabetes worries, and increased disclosure of T2D related worry/distress predicted receiving higher levels of spousal support (Choi et al., 2020). Overall, limited studies focus on the role of adult children supporting aging immigrant parents with T2D.

Most studies of Whites with T2D have examined family support through the lens of spousal support (Albanese et al., 2019). For non-white families, the adult child may be a more important source of support for the aging parent (Rote and Moon, 2016). Older Asian Americans are mostly foreign-born immigrants (Budiman & Ruiz, 2021). Studies of older Asian immigrants have shown they face hardships in terms of health care access, limited English language proficiency, and knowledge deficiency on supportive resources (Wu & Qi, 2022). Because of their limited English proficiency, poor health literacy is widespread among older Asian American immigrants who live with chronic health conditions (Chang, 2019), resulting in poorer outcomes for diabetes (Leung et al., 2018).

As a result, older Asian American immigrants with T2D may rely on their more acculturated and English-speaking adult children. Adult children of Asian immigrants often serve as language and cultural brokers in health care settings, thereby increasing health literacy and improving health outcomes (Yoo & Kim, 2014). In addition to the barriers that older Asian American immigrants may face that adult children help with, adult children’s caregiving roles may also be influenced by culture: filial piety, a value of Confucianism, emphasizes responsibility to parents including respect, obedience, and provision of emotional and financial care and support (Lim et al., 2022). Because of these various reasons, children can play the primary role for medical information and diabetes management by supporting and educating their parents and advocating on their behalf in medical settings (Doan et al., 2023). Other studies reported that training adolescent children of patients with T2D not only improved diabetes prevention amongst the children, but also assisted in their parents' diabetes self-management program (Gefter et al., 2016). Additional studies have shown that adult children can help patients access and use health information technology and assist with diabetes management (Nelson et al., 2018; Seng et al., 2023).

### Family Caregiving Trajectories

Previous research describes prototypical trajectories of caregiving patterns between care recipients and their informal, familial caregivers i.e., spouses and adult children (Schulz et al., 2020). Caregivers begin this longitudinal trajectory by experiencing increased awareness of the care recipient’s changing health status which may necessitate limited assistance with health-related tasks (i.e., joining medical appointments, monitoring functioning). Over time, care recipients may have increased care needs including assistance with daily household tasks, monitoring health symptoms and medications, hiring advanced care providers and coordinating care, emotional support, and self-care (i.e., dressing and mobility). At the final stage of this longitudinal trajectory, informal caregivers may initiate end-of-life care, including placements in long-term or hospice care facilities. Caregiving in this trajectory is cumulative, such that as the care recipient’s health condition progresses (i.e. increased disability, need for care), the caregiver’s roles and responsibilities increase and become more complex.

Given increased risk for diabetes, adult children of older Asian American immigrants can be a pivotal source for family support in promoting self-management of T2D. However, few studies exist that examine the caregiving trajectories and roles of adult children in improving health literacy and providing support to older parents with T2D. Thus, the purpose of this paper is to explore trajectories of family support dynamics between older Filipino and Chinese American immigrants with Type 2 Diabetes and their adult child.

## Methods

### Sample Characteristics

This paper is part of a larger mixed-method study which explored family support dynamics of older Asian Americans with T2D and their adult children. The larger study included a quantitative survey that assessed factors for family support among dyads of an older AA with T2D and their adult child without diabetes, including demographic characteristics, acculturation, health literacy, diabetes knowledge, and depression. This paper focuses on a sub-sample of 10 dyads consisting of older Chinese American and Filipino American immigrants with T2D and their adult child. Dyads participated in qualitative, in-depth, face-to-face interviews. Because dyadic methods are expected to yield rich data, 10 dyads with individual and dyadic interviews were selected as the point of saturation. A total of 30 interviews were conducted, including 20 individual interviews and 10 dyadic interviews (between and with) the older adults and adult children (Table 1).

**Table 1:** Characteristics of Chinese American & Filipino American parent-adult child dyads (n = 20)

*Variables*	*Overall* *(n* = *20)*	*Parents* + *T2D* *(n* = *10)*	*Adult Child* *(n* = *10)*
Gender			
Female	14 (70%)	7 (70%)	7 (70%)
Male	6 (30%)	3 (30%)	3 (30%)
Ethnicity			
Chinese	10 (50%)	5 (25%)	5 (25%)
Filipino	10 (50%)	5 (25%)	5 (25%)
Mean Age Years (SD)	48.15 (± 18.3)	64.3 (± 9.1)	32 (± 6.7)
Marital Status			
Never Married/Single	5 (25%)	0 (0%)	5 (50%)
Married/Partnered	14 (70%)	9 (90%)	5 (50%)
Divorced	1 (5%)	1 (10%)	0 (0%)
Widowed	0 (0%)	0 (0%)	0 (0%)
Years in the US			
Native Born	7 (35%)	0 (0%)	7 (70%)
≥ 10 years	10 (50%)	9 (90%)	1 (10%)
5–9 years	2 (10%)	0 (10%)	2 (20%)
< 5 years	1 (5%)	1 (10%)	0 (0%)
Spoken English Ability			
Very Well	12 (60%)	5 (50%)	7 (70%)
Well	4 (20%)	3 (30%)	1 (10%)
Not Well	3 (15%)	1 (10%)	2 (20%)
Not at All	1 (5%)	1 (10%)	0 (0%)

*SD* Standard Deviation

The inclusion/exclusion criteria for this sub-sample consisted of the following:

### Inclusion/Exclusion Criteria of the Study

#### Parent

Self-Identified as Chinese or Filipino, 45 ≥ years, diagnosed with T2D, first generation immigrant, a California resident, and English or Chinese literate.

#### Adult Child

Self-Identified as Chinese or Filipino, 18 ≥ years, not diagnosed with T2D, California resident, English literate, has one parent with T2D, and has contacted (e.g. phone, text, email) or visited parent in the past 6 months.

### Procedures

Institutional Review Board approval was obtained from San Francisco State University. Participants were recruited through San Francisco (SF) Bay Area community organization meetings and events, word of mouth, and flyers (Doan et al., 2023; Peregrina et al., 2022). Dyads who met inclusion criteria were recruited from the larger pool of survey participants. Upon completion of surveys, dyads were invited to participate in a follow-up, interview study. To observe interpersonal processes, interactions, and communications between Chinese American and Filipino American older adults and their adult child, dyads were interviewed on ZOOM individually and together regarding T2D. Interviews with individuals were 30 min each and dyadic interviews lasted approximately 60 min. Informed consent was obtained prior to study participation. Each participant received a $50 online gift card in appreciation for their time. Interviews included the following topics: role(s) within family before and after T2D diagnosis, T2D knowledge, challenges and burdens of parent diagnosis and treatment, concerns/worries about diagnosis/treatment of parent with T2D, experience of social interactions with family and non-family regarding diagnosis/treatment of T2D, and experiences regarding social support for parent with T2D.

### Data Analysis

All ZOOM interviews were digitally recorded and transcribed. All ZOOM interviews were also transcribed by a trained transcriptionist. A three-step process was used in conducting qualitative data analysis. First, four different trained coders reviewed transcripts independently to identify themes and coding categories. Second, transcripts were then coded for themes that ran consistently throughout interviews. Qualitative analysis experts (HP, GY) oversaw the analysis. Third, all authors met to review the codes again. The field notes included detailed descriptions of the following observations and issues that arose during the interviews: interviewer’s impressions of major themes from the interview, interactional style and rapport between interviewer and participant, communication style and language use, and observations of emotional affect. In the first iteration of qualitative analysis, the data was coded using grounded theory analytical methods (Corbin & Strauss, 1994) to identify themes, trends, and patterns across interviews and in field notes. Two trained researchers coded interviews in parallel to ensure an inter-rater code reliability threshold of 0.80, as determined by the Cohen’s Kappa coefficient (McHugh, 2012). The 30 interview transcripts were examined to determine whether the content of each item is relevant.

## Results

Four major themes emerged in interviews (N = 30) with individual parents and adult children separately, as well as the dyadic interviews with both parent and child. A key theme throughout is that family support often followed a trajectory around the needs of parents with T2D. The trajectory of family support occurs in stages, where, as parents’ health status change, adult children experience increased awareness of the parents’ health status, increased desire and need to be involved in care, and more accumulated caregiving responsibilities. The themes around family support illustrated that support may progress through stages including: independence, transitions, partnership, and stepping up. These themes around family support followed a collective family trajectory centered on increased recognition of the seriousness of the parent’s Type 2 Diabetes and increased involvement of adult children (Table 2).

**Table 2:** Main Themes: Stages of Support in the Dyad

Theme	Dyadic Definition	Parent Perspectives + Action	Adult Child Perspectives + Action
Independence	The Parent is managing Type 2 Diabetes on their own terms and networks No support given by the child for Type 2 Diabetes management	I can manage Type 2 Diabetes on my own or with my spouse	There are no physical disabilities, so my parent does not need my support
Transitions	Growing concern by adult child Reluctance by parent on receiving support Increasing tensions in the dyad	I want to continue to be in charge of my health I don’t see the need for my child to help with my Type 2 Diabetes	I see a growing need to support my parent There are growing tensions and I am unable to figure out how to support my parent with Type 2 Diabetes
Partnership	Gradual need for support from adult child (e.g. food, information) in terms of managing Type 2 Diabetes in older parent	I will accept help from my child in only some areas of my health	I understand that we must navigate T2D as a family and want to provide support, but food/exercise are the only way(s) I know how
Stepping In	Adult child supports or takes over the management of Type 2 Diabetes for the older parent (e.g., needing to go w/ parent to doctors’ appointments, checking blood sugar, preparing meals, etc.)	I need all the support I can get to manage my Type 2 Diabetes and other comorbidities and my child can help	I must provide support because my parent can’t manage anymore

## Independence

“Independence” is the first theme among the dyads. This theme represented the aging parent who felt they could manage T2D on their own or, if married, with their spouse. The adult child viewed their aging parent as “independent” who does not need support. For these dyads, the aging parent focused on understanding and managing the disease process on their own, while the adult child was not aware or did not recognize any immediate need for support of their older parent with T2D. Moreover, the adult child did not see the severity or complications of the disease process, or even knew that their aging parent had a T2D diagnosis. There were no discussions on how to elicit support from the adult child and as a result, adult children felt they did not need to help. Rather, the focus in these adult child-parent dyads was that of the aging parent managing food and lifestyle changes on their own or with their spouse (if applicable), rather than rely upon their adult child for T2D management.

## Ken and Olivia: Adult Son and Mother

Olivia and Ken, a Filipino American parent–child dyad, is an example of the older parent managing Type 2 Diabetes without the support of their adult child. Olivia is retired and is 74 years old and lives with her husband (Ken’s stepfather), who is her primary support with T2D management. Ken is the middle child and is 42 years old. He lives almost 500 miles away and Ken’s two other siblings also live at least 300 miles away from Olivia. Olivia and Ken communicate primarily through text, email, and phone calls.

Olivia has lived with Type 2 Diabetes for 4 years and lives with multiple chronic health conditions. She recognizes that management of T2D means that she must do regular tests, exercise, and make changes to her diet. As her father had T2D and her siblings currently live with it, she is aware of her various health challenges and the seriousness of her T2D. She also is aware of the tremendous lifestyle change that is needed:

Well, diet, exercise, medication and really a change of lifestyle. Yeah, because we were raised to always have dessert after each meal. I mean especially lunch and dinner. So now instead...and I love chocolate...that's been hard for me. I like chocolate candies...I still eat it, but not as much as I used to, and we eat more fresh fruits. I used to love to eat ice cream…I used to love to drink Pepsi and all that, I stopped doing that… I'd rather stay with tea and water.

Olivia’s focus is managing T2D through changing how she eats and staying active. Her husband is her sole support, who reminds her to not overeat, and walks or goes dancing with her. She does not rely on any of her adult children. When asked, “How do you support each other as a family?” Ken answers, *“I don't do a very good job at all. I don't actually… I don't… I don't do anything at all.”* Ken states that his mother has never really spoken about her T2D. He also states that they live in different cities, and he is not sure if physically, he could be of support, due to the distance and his busy work schedule. Ken and Olivia’s experience may demonstrate structural constraints (i.e., necessity to work and live at a long distance) on adult children’s capacity to provide tangible care for their parents, thus placing parents in positions to exert independence over T2D care.

When asked if anything has changed as a result of his mother’s diagnosis, he states, *“No, not at all.”* Ken continues to discuss that his siblings are spread across the US right now and as of the moment, they do not feel an urgency to help, since his mother does not have physical disabilities that would warrant more concern about her illness. He states: *“I definitely think my parents are in charge* (of Olivia’s diabetes) *…I don't think either… parent actually have any physical disabilities. So there isn't much to do.”* His mother echoes a similar sentiment about her management of T2D: *“I can manage. We don’t talk about it.”* This dyad demonstrated the theme of independence, that support was not warranted for an adult child to get involved with the aging parent. At this point, everyone is too busy. The parent can manage T2D independently on her own and her husband supports her.

## Jillian and Miranda: Adult Daughter and Mother

Jillian, a 25-year-old Filipino American daughter and her 63-year-old mother with T2D, Miranda, represent the older parent’s independence in managing T2D. Miranda has had T2D for 25 years and is separated from her husband. Both work full-time and reside together in a single-family home.

Jillian describes her relationship with her mother as “surface level” and they do not have a close emotional relationship. She has never been asked to take a caregiving role for her mother. She discussed that food was how she tries to support her mother’s T2D management. *“The few conversations we have around food happen during the times we are already out of the house and decide to eat out; those conversations usually consist of statements and questions along the lines of: ‘What can we eat that's healthy?”* When asked if she has helped with her mom's healthcare, like doctors’ visits or other types of care, she states:

No, I think that those are very...private appointments. I don't necessarily join them. I'll drop her off if she needs a ride, but that's pretty much it...since I’m not sure how the process works. I think it's all mutual because my mom has more understanding of her health. And then what she can eat and can’t eat. So, she's in charge of what she’s eating.

This dyad holds a mutual understanding that the aging parent has knowledge of their health condition and can manage on their own. This dyad thus represents independence and demonstrates boundaries in the relationship where the aging parent expects to be allowed to manage T2D on their own without tangible support from their adult child.

## Transitions

In “Transitions,” the dyads were in the midst of transitions of the adult child becoming more aware of their parent’s needs and the parent being reticent about this awareness. This theme was characterized around negotiations and tensions around the parent’s T2D. Parents wanted their independence and to maintain the primary caregiver role in the family. Adult children knew that diet and exercise were important in their parents’ management and sought ways to support this matter. This theme was marked by collective tensions, parental allowances, the adult child’s worries and ultimately, a shared understanding that diabetes was primarily managed by the parent at this time.

Parents in this stage expressed desires to avoid relying on their adult children. Tensions arose in dyads when adult children reflected on the need to be more involved in their parents’ health, support that was dismissed by parents. Parents in this stage allowed their diabetes to be managed on a family basis (only through diet and exercise), while still maintaining their role as the primary caregiver for their families. While adult children were growing in their awareness of their parents’ health condition, adult children were not yet going to doctor visits or involved with more in-depth care and concerns. Two dyads reflected tension and growth towards shared understandings.

## Charlotte and Ava: Adult Daughter and Mother

Ava, 59, is a retired, single Chinese American mother with two daughters and a son. Ava spends much of her time socializing at a local senior center, where she regularly obtains her meals and participates in exercise classes. Ava considers her diabetes management as proactive. Although Ava reports that she has an ideal relationship with her daughter Charlotte, who is 25 years old, her daughter reflects on her family’s disagreements over their mother’s diabetes management.

Charlotte describes that tensions have arisen because she wants to be more involved in her mother’s care but is met with disagreement. Charlotte states:

It’s a bit stressful because I think the way my mom and I approach health is…different sometimes. She is pretty proactive…doing all these activities, but I have disagreements with her over nutrition and how to diet. So I can sometimes cause a little bit of tension...So maybe it’s just more like a desire for control…but I think my mom doesn’t want to depend on me too.

In addition, just talking about managing diabetes brings up disagreements and *“these are…swept under the rug.”* Evelyn wishes that she could be more involved in her mother’s care, especially in nutrition, and Ava’s relationship and communication with her physician.

## Sophia and Mia: Adult Daughter and Mother

Mia, 58, is a Chinese American mother and Sophia, 26, her daughter and only child, describe the tensions and challenges that they face as they navigate caregiving roles within their families. Mia provides care for her aging in-laws, and explains her reluctance to seek support from family:

I helped them cook and do laundry instead of being helped by them. Because my parents-in-law are getting very old. Every week, I have to go…shopping for them also. I have to take care of two families.

Although Mia maintains her role as the main care provider in the family, Sophia, reflects on her desire to take a more active role in her mother’s health. Sophia describes her lack of knowledge regarding her mother’s condition due to the lack of communication and the secrecy that her parents have had in minimizing diabetes in the family, which she considers cultural. She discusses her frustration:

I feel like they’ve been protecting me. I feel like sometimes they make it sound not as scary as it seems. I think they don’t want me to be worried…unless it’s…really bad, like with my dad, (he) needed to go to the hospital.”

Despite her lack of understanding of the full scope of her mother’s diabetes diagnosis, Sophia shares the ways that she has tried to be involved in her mother’s care, by encouraging her mother and family to go on walks more and making dietary changes.

These two dyads exemplify how there are tensions around the parent’s perception that they are managing their diabetes and do not need their child’s support, but the adult child’s worry that their parent is not managing.

## Partnership

In “Partnership,” parents allow their adult children to support their management of T2D. One of the first ways adult children partner and support their aging parents with T2D is by discussing the condition with them and teaching them about healthy eating and exercise habits. Aging parents, who may have relied on themselves or their spouse to manage their health-related behaviors, begin to allow their adult child to support them with eating healthier and exercising. Adult children and aging parents in this phase have a shared understanding that they must navigate T2D as a family.

## Ella and Camila: Adult Daughter and Mother

Ella, age 40, a Filipino American, was not aware of her mother’s diagnosis until recently. Camila, 67, has self-managed T2D for 23 years. Camila’s husband was first diagnosed with T2D and comorbidities, therefore his health needs became a focus of the family’s concerns. Ella has been able to support both of her parents with T2D through healthy eating, despite their physical distance. Ella reflects on the barriers that her family has experienced in regulating diet (particularly Filipino food), cultural values and family gatherings:

That aspect…the wastefulness around food…Filipinos like valuing food and that (food is)…a language of love and then…me coming in and just throwing it all away. I think that was a very hard thing. Food is often seen as a gesture of love and care in Filipino culture.

The other thing culturally around food would be our gatherings. And I think that's less about me. We have the side of our family heavy into Filipino food, but even just the idea, the gathering revolves around food, and...to restrict what you bring and the quantity would be less love.

For Ella and Camila, Filipino traditions around family gatherings and the symbolic meaning of food pose as barriers to proper T2D maintenance. These barriers are managed through strategic efforts to limit food intake, which at times, may be seen negatively as denying the family’s love and care.

When asked how else they support each other as a family with members who have T2D, both Ella and Camila mentioned exercising together, monitoring blood glucose levels, and Camila’s son helping to cook healthier foods. As a family, they would often go to the gym together, and join classes such as Zumba or Senior Fit. During their regular visits, Ella also prioritizes taking walks with her parents instead of driving. In addition to these roles, Ella also monitors her parents' fitness and blood glucose levels.

Because like I said, I spy. I know they both write down their blood sugars in the bathroom. I always check it. And their numbers are good. But…it would be helpful to know what the advice is that the doctor’s saying, how are they (both parents)? How is he (the doctor) presenting it to them (regarding) their health? I think she (Camila) also doesn't want us to worry.

Besides diet and exercise, Ella is now focusing on finding ways to be more proactive in her parent’s health *“I think what's helpful for us is having that open communication and…how can I be more involved with their doctor visits? That might be something that I definitely* (will)* try to do more. They're not so sick yet. Why do we have to wait for that?”.*

Camila reflects that by observing her children’s healthy lifestyle, Camila has become more conscious about living a healthier lifestyle.

Because Ella and her family eat very healthy… maybe translate that more. Not just for when we're here, but also when we're home. But then again...since my son's back home and he's more health-conscious with the food, so that helps. It’s hard for my husband and I. We're transitioning, but it is just hard to break old habits.

With her daughter’s support, since her retirement, Camila has become more cognizant of the ways that her body is changing, prompting her to designate time to prepare healthy meals and exercise. This dyad thus represents partnership, where the aging parent accepts support from their adult child, albeit support that is limited to diet and exercise.

## Natalie and Carter: Adult Daughter and Father

Carter, a Filipino American, age 73, was diagnosed with diabetes almost 2 years prior. He and daughter Natalie, age 36, have open communication around finances, work, and family matters. Both father and daughter describe navigating Type 2 Diabetes together as a family. Natalie recalls that in the past, they never talked about health and that she respected her parents’ need for privacy. Carter does not openly share deep personal, emotional, or health-related information with Natalie, unless she explicitly asks. It was an eye-opener that her parents were getting old when they started talking about health and the need to manage Carter’s T2D.

Carter states that he is now open about sharing his different health conditions with his family and describes the support he receives from his daughter through reminders of eating better and exercising. His wife is his main champion for daily reminders regarding his diabetes management and his companion when exercising. Carter recognizes their support and his own stubbornness:

I am hard headed. I love…Filipino food. I’m always bothered by my wife every day on what kind of food I eat.... My daughter is always reminding me about eating vegetables and to do some exercises…(they) asked me to bring the kids to school, walking, so that I can have exercise. If I really want to live, I have to be consistent now...So I'm working on my exercise and sometimes I reduce eating out and just eat at home so I will not eat a lot of those foods, kind of remove the temptation. If you eat out...especially a buffet style of eating, then you are tempted to eat those things that I should not be eating. That’s how we do it.

His wife does the cooking for maximum observance of the diet, while Natalie said she asks him daily, “*Have you been eating well?” How's your blood pressure?”* She also said she follows up on her father’s health. His daughter called her support “nudges.” Natalie states:

I think my dad, you know, you kind of have to nudge him a little. We do a little checks and balances for each other like, ‘Okay, maybe you want to slow down on ...a little bit of the rice. I think that's enough cake.’ Also just me being there as a resource if they need it. Pretty much just being there if they have questions. I could probably do better and be more proactive. I could change, you know...reaching out more.

Both dyads illustrate how the adult child navigates supporting their aging parent with T2D, through various nudges regarding blood sugar, diet, and exercise. The reminders and role-modeling are received by the parent as support.

## Stepping In

The last theme, “stepping in,” involves the parent and their adult child recognizing the importance of familial support to manage T2D. At this stage of stepping in, the aging parent recognized the need to involve their child in managing their health conditions and were willing to accept support. Parents at this stage experienced significant progressions in their physical health condition. Often, these aging parents were also dealing with additional chronic illnesses and/or may have had a hospitalization or catastrophic event that may have warranted the need for adult children to get involved. Until this time, most children relied on their parents to manage T2D on their own, with support limited to food and exercise information. In this stage, the adult child stepped in to actively support their parent’s healthcare (i.e. attending doctor’s visits, medication adherence, diet, and exercise).

## Luke and Henry: Adult Son and Father

Henry, 81, a Filipino American, has lived with T2D for 25 years. He is retired and lives with his wife. They have three grown children. Their daughter, who helps with their household and healthcare needs, lives ten minutes away. Among their two sons, one lives out of state and the other, Luke, lives 70 miles away. Luke is 40 years-old and visits his father one to two times a month, or more frequently, when health emergencies arise for both Henry and his wife.

Luke is an example of an adult child stepping in, not only helping a parent manage T2D, but also involving themselves in their parents’ health care. Luke mentioned that in the early years of his father’s diagnosis, Henry was able to manage his T2D. Luke said that over the years he noticed changes in his father:

I've definitely noticed a change in timing right there. A little bit slower to respond, to comprehend, to act on any other things. So...eating...takes a little bit longer. Taking care of their daily chores takes a little bit longer. Managing healthcare...it takes a lot more assistance. I've noticed that directly. I've noticed how it impacts them mentally...there's a little bit of madness that it's not purely self-control.

When asked how he and his sister became involved in supporting their dad’s T2D, Luke mentioned a car accident two years prior, in which his dad fell asleep at the wheel, that T2D played a role in his father’s car accident. As a result of the car accident, Luke moved from the East Coast to come and support his parents. He states:

My father was in a car accident. That's when everyone stepped up in making sure we are aware of...how to maintain Dad’s health…and understanding what the recovery was going to be like, and the health systems…In terms of his T2D, we do make sure he follows his daily log. What time he has to take his blood sugar tests in the morning, mid-day, and at night.

Luke describes facilitating communication and understanding between his father and medical providers during doctor's visits. He states that communication with his dad’s doctors has become one-way since his Dad’s accident. Luke’s role has been to step in and listen, and help his father understand: “*Me listening, and just repeating, and making sure that whatever the doctor said resonates with my father, or that I understand what the doctor (is saying).*”

## Chloe and Violet: Adult Daughter and Mother

Violet is a 63-year-old Chinese American mother, and her daughter Chloe is an only child. Violet was diagnosed with T2D approximately 23 years ago. Like many individuals, she at first was in denial, not wanting to admit she had T2D, and rejecting lifestyle changes.

Although the beginning of her fight against T2D was filled with insecurity and doubt, Violet began to feel supported in her efforts to manage T2D because of her daughter’s support. Chloe stepped up to support her mother’s T2D by visiting weekly, attending doctor’s visits, and monitoring her mother’s diet. Violet reflects on how she felt about her T2D being out of her control:

When I first was diagnosed, I was in denial. I needed someone around me, like my family to help me fully understand that this is a controllable disease, and I don’t need to be depressed…My daughter and my husband, they were key to my being successful in managing my diabetes because they are very supportive. They truly understand what this disease is about too.

With the help of her husband and daughter, Violet’s perspective changed, and she recognized that T2D is controllable. She also became more open to adjustments in diet and exercise, even going out of her way to take classes at Kaiser to further understand what T2D is and what are the best approaches.

So they…would tell me, why I cannot have porridge. My husband and my daughter are both very supportive for my health condition. We…exercise (together), and you need to stay away from those (food) that are very high in carbohydrates. And…my daughter helped…control my…condition.

Because of their family dynamic, both Violet and Chloe feel confident managing Violet’s T2D. Their awareness and emotional support help carry much of the mental and emotional burden that comes with having T2D. With this load lifted, the parent can focus more on their physical lifestyle changes rather than the emotional battle with T2D.

## Easton and Hazel: Adult Son and Mother

Hazel, a Chinese American, is 50 years old and has lived with diabetes for almost 4 years. Along with diabetes, she has other pre-existing conditions including high blood pressure and cholesterol. Her son Easton is 25 years old and lives at home. Both Easton and his father are Hazel’s primary caregivers who oversee her workload and manage her diet. Easton reflects on how he has stepped up to support his mother:

My father takes my mom to see a doctor, since I am a little bit busy because (I) need to go to school. I have a part time job. Usually I don’t take my mom to see a doctor, but usually I will pick up the medicines for my mom from Walgreens, and mostly I just take care of what my mom eats.

In order to balance their school and work commitments, Easton and his father work together to manage Hazel’s diabetes. Hazel is the only member of her family who has multiple comorbidities. Her husband, who also has a history of T2D, accompanies her to appointments to support communication with the doctor. Despite having a busy schedule, Easton realizes that the pain his mother is feeling, along with the stress of work, may be overbearing. As her main source of support, Easton’s caregiving responsibilities include preparation of food, and research to help manage his mother’s health condition. He takes time out of every day to talk and share meals. Despite his limited knowledge of T2D, he puts in the effort by researching different methods to help keep his mother’s many chronic illnesses under control. He says:

I will spend my free time preparing food for my mom. Since she has (a) lot of stress from the job, she might talk about what’s the problem in her job. I am a student in school, I will talk about something about my class. Also share what I’ve seen interesting (on) the internet.

In recent years, Easton and his father noticed the physical toll that T2D had taken on Hazel. Although she tries to endure her pain from her T2D, she does not have as much energy as before. Due to lack of energy and consistent fatigue, she sleeps more often and for longer periods of time. Hazel reports:

They are very understanding. They said, ‘okay if you want to sleep just go back to sleep.’ They are always very understanding. ‘No problem, don’t worry, just do whatever you want.’ So they do shopping, cleaning and cooking.

Both Hazel and Easton reflect that they have an “ideal” parent and child relationship. With support from her son and husband, Hazel has learned to manage her condition. ***“****For me right now, my son and my husband, they are perfect. And I can see I am getting better.”* Through support from her son, particularly in diet, exercise, and medication management, Hazel has learned to manage her multiple comorbidities and feels confident in living a healthier lifestyle.

## Ruby and Luis: Adult Daughter and Father

The last Chinese dyad of Luis and Ruby, describe a harmonious relationship between an aging parent and his daughter when managing T2D. Luis is 57 years old and was diagnosed with T2D five years ago. Ruby, his only child, is 32 years old. She remembers the difficulty at the beginning of Luis’s diagnosis, that they *“faced T2D together,”* and stepping up to support him through, *“applying for health insurance and reminding him of taking medications…We looked for information about what is good or bad for his T2D and tried to know more about it.”*

Because they live together, Luis and Ruby talk to each other daily. Luis says that he shares everything with his daughter and that *“she takes care of me.”* He continued and described how Ruby *“manage*(d) *my diet, urged me* (to) *exercise and reminded me to take medications on time.”* Also, his family sometimes joins his daily 30-min walks.

Ruby supports her father’s T2D management. *“If he eats unhealthy foods, I will stop him from eating. We care about food a lot, and often discuss how to eat healthily.”* Ruby also attends her father’s health appointments, saying:

We always go together because my dad needs help understanding what the doctors say. We can understand most of what the doctors say but we have difficulty when it comes to some professional terms.

Ruby has stepped up with supporting Luis manage his T2D through diet, exercise, and attending doctor appointments. Luis accepts his daughter’s help and feels they have a mutually supportive and ideal parent–child relationship.

The four dyads above describe the adult child “stepping up” even more, to help their aging parent manage T2D. Dyads describe the adult child’s role in supporting the parent regarding diet and exercise, making sure blood sugar checks are done, participating in appointments with healthcare providers, and maintaining the household. Additionally, adult children provide emotional support. The aging parent accepts the contributions of their child because they are now unable to manage T2D by themself.

## Discussion

Our qualitative findings describe a trajectory of stages where family support dynamics gradually shift to allow adult children to take roles in their aging parents T2D management, while parents learn to accept this support. The stages include: independence, transitions, partnership, and stepping in. At the Stage of “Independence,” adult children are not involved in their parents’ management of T2D, and parents manage T2D on their own or with a spouse. At the Stage of “Transitions,” adult children navigate negotiations and tensions with their parents around T2D management. In this stage, the parent does not see the need for help from their child and wants to continue managing their own health while children may seek greater involvement in their aging parents’ health. At the Stage of “Partnership,” adult children directly support their parents by promoting healthy diet and exercise, and the parent is accepting of this limited help. Finally at the Stage of “Stepping in,” adult children play active roles in supporting their parents’ healthcare, while parents recognize and accept help from their children. Families progress through the stages depending on the parents’ health and the family relationship.

Multiple factors may explain the family support dynamics found between Asian American parents diagnosed with T2D and their adult children. Previous studies report that Asian American older adults have shifting and changing meanings of family support that is more about self-sufficiency and independence and, if needed, adult children are often the source of support (Wong et al., 2006). This shifting meaning may be a reason why receiving social support from adult children is a process for Asian American families, as many aging parents may want to manage their own health independently before relying on their adult children. At the same time, adult children in this study showed concern throughout the various stages and were ready to serve as an advocate for their parents with T2D. Yoo and Kim (2014) found that adult children of Asian immigrants often serve as language and cultural brokers for their aging parents as they face declining health. Filial piety may also play a role in caregiving dynamics in Chinese and Filipino American families, as children seek to demonstrate respect and care for their aging parents through concerns about their parents’ changing health (Doan et al., 2023).

While self-management of T2D is rooted in the individual, studies have shown that family members play crucial supportive roles in managing diabetes of a loved one (Thirsk & Schick-Makaroff, 2021). Increased familial support has been shown to decrease diabetes-related distress (Young, et al., 2020). Thirsk and Schick-Makaroff (2021) have found that framing of a health issue, whether it be individual or familial, can impact chronic disease management and the provision of family support. Thus, framing a family member’s diagnosis as a collective, familial issue may elicit more family support and a collective and collaborative response to managing aging parents’ T2D. An important outcome for family diabetes interventions among Asian American families might be working to frame a diagnosis as a family issue which could increase supportive family interaction patterns and decrease obstructive behaviors. Very little research has been written on family appraisals of chronic diseases among aging family members. In addition, research on family support for T2D has primarily utilized White, Black, or Latino families. This paper provides a contribution as it examines adult children and their parents that are Chinese American and Filipino American immigrants.

## Limitations

This study has several limitations to be considered. Due to the limited number of participants, our results are not transferable to the whole population of Chinese and Filipino American families with T2D. Furthermore, our study design is cross-sectional in nature, and reflects dyads’ reflections of their caregiving dynamics within a given point in time. Future studies can utilize longitudinal designs to assess how support between an adult child and an aging parent may shift over time. Despite these limitations, this is the first study to explore family support dynamics among Filipino and Chinese American families with a parent diagnosed with T2D. Our study is unique in exploring the needs and perspectives of both the aging parent and their adult child caregiver.

## Conclusion

Our work demonstrates that aging parent and adult child perspectives around autonomy and illness are often associated with family tension, support, and self-management of T2D. Many aging immigrants with T2D wanted to remain as independent as possible and not rely on their adult children. Over time, adult children may feel propelled to be healthcare brokers for their aging parents by providing language assistance, mediating healthcare institutions, and educating parents on how to best manage their T2D. Our research demonstrates parent–child dyads may initially disagree about autonomy and interdependence, and gradually transition to allowing children to fully support their aging parents’ health condition. Within the four stages of the trajectory, aging parents gradually learn to accept and recognize their need for family support, while adult children also learn to accept their parent’s T2D while balancing multiple roles. Limited research focuses on family attitudes around autonomy and illness. Continued research is needed to understand how families work intergenerationally to support the management of an aging parent’s chronic health condition.

## Data Availability

Due to the nature of this research, participants of this study did not agree for their data to be shared publicly, so supporting data is not available.

## Code Availability

**N/A**.
